# Elevated Corpus Callosum T1rho Reflects Disease Burden and Structural Atrophy in Multiple Sclerosis

**DOI:** 10.3390/diagnostics16152368

**Published:** 2026-07-28

**Authors:** Lei Wang, Tiffany Y. So, Ziqiang Yu, Weitian Chen, Joseph C. H. Choi, Qiyong H. Ai, Queenie Chan, Alexander Y. L. Lau

**Affiliations:** 1 Department of Imaging and Interventional Radiology, The Chinese University of Hong Kong, Prince of Wales Hospital, Hong Kong SAR, China; 1155104675@link.cuhk.edu.hk (L.W.); ziqiangyu@cuhk.edu.hk (Z.Y.); wtchen@cuhk.edu.hk (W.C.); hemisai@cuhk.edu.hk (Q.H.A.); 2 Division of Neurology, Department of Medicine and Therapeutics, Faculty of Medicine, The Chinese University of Hong Kong, Hong Kong SAR, China; ccho19921209@gmail.com (J.C.H.C.); alexlau@cuhk.edu.hk (A.Y.L.L.); 3 Philips Healthcare, Hong Kong SAR, China; queenie.chan@philips.com

**Keywords:** multiple sclerosis, T1rho imaging, corpus callosum, corpus callosum index

## Abstract

**Background/Objectives:** Corpus callosum atrophy is a well recognized feature of multiple sclerosis (MS), which has been associated with disease duration and severity. Quantitative T1rho imaging is an MRI technique sensitive to microstructural tissue alterations, but the T1rho characteristics of the corpus callosum in MS remain largely unexplored. This study aimed to characterize T1rho in the corpus callosum in MS patients, and examine relationships between T1rho values, callosal atrophy, and clinical and imaging markers of disease burden. **Method:** Thirty patients with relapsing-remitting multiple sclerosis (RRMS) and 30 healthy controls underwent T1rho imaging. T1rho values were measured in the genu, body, splenium, and whole corpus callosum. Comparisons between patient and control groups were performed using the Mann–Whitney U test and the independent samples *t*-test, and Pearson’s and Spearman’s correlation coefficients were used to evaluate the correlation between imaging and clinical measures. **Results:** T1rho values were significantly increased across all corpus callosum subregions in patients compared to controls. Corpus callosum T1rho values showed correlations with disease duration (r = 0.375–0.408, *p* < 0.041), lesion load (r = 0.726–0.810, *p* < 0.001), and the corpus callosum index (CCI) (r = −0.731–−0.640, *p* < 0.001). **Conclusions:** T1rho values are elevated in the corpus callosum of MS patients and associated with disease duration, lesion load, and callosal atrophy.

## 1. Introduction

Multiple sclerosis (MS) is an immune-mediated disorder of the central nervous system [1], characterized by diverse clinical manifestations and variable courses of disease progression [2]. Relapsing-remitting multiple MS (RRMS), the most common disease phenotype, is characterized by clearly defined episodes of acute neurological dysfunction followed by periods of partial or complete clinical recovery [3,4]. The corpus callosum, the largest commissural fiber tract in the brain [5], is frequently affected in MS, with callosal atrophy being a well-recognized feature [6]. Histopathological study has demonstrated evidence of axonal loss and reduced fiber density within the corpus callosum of patients with RRMS, suggesting the presence of progressive underlying neurodegenerative changes, despite the episodic nature of clinical relapses [7].

Research indicates that corpus callosum atrophy may be associated with MS disease duration and severity [8]. The corpus callosal area has also been correlated with cognitive deficits, including impairments in processing speed, composite memory, and executive dysfunction [9]. The corpus callosum index (CCI) has been introduced as a simple neuroimaging measure for assessing callosal atrophy [10], derived from normalized callosal thickness measurements on midsagittal MRI slices [10, 11].

Quantitative T1rho imaging is an emerging technique that assesses tissue macromolecular content by characterizing spin-lattice relaxation in the rotating frame [12]. Its sensitivity to low-frequency interactions, particularly between protons and macromolecules [13], makes it a promising tool for detecting early pathological changes in the brain [14,15]. In contrast to T1 relaxation, which reflects motion at the Larmor frequency, and T2 relaxation, which is sensitive to interactions with static local magnetic fields, spin-lock radiofrequency (RF) pulses probe molecular motions occurring close to the spin-lock (SL) frequency [13]. T1rho has shown excellent reproducibility across serial scans, allowing even subtle variations to be reliably interpreted as biopathological changes [16]. Several studies have documented T1rho alterations in MS lesions and the normal-appearing white matter (NAWM) and suggested that T1rho can detect not only overt demyelinating lesions but also more subtle changes in tissue that appears otherwise normal on conventional MRI [14,17,18]. However, while previous research has predominantly characterized T1rho changes within the global white matter, the specific T1rho characteristics within the corpus callosum remain largely unexplored.

This study aimed to: (1) characterize T1rho relaxation properties in the corpus callosum in patients with RRMS and compare them with healthy controls; (2) assess the corpus callosum using the CCI as an index of callosal size and atrophy; and (3) examine the relationships between T1rho values, CCI, and clinical measures of disease burden and disability, including disease duration, Expanded Disability Status Scale (EDSS), and lesion burden on MRI.

## 2. Materials and Methods

### 2.1. Study Participants

This study was approved by the local institutional ethics committee (2020. 650). Each participant provided written informed consent before joining the study. A total of 30 patients aged 18–55 years with a diagnosis of RRMS according to the revised McDonald criteria [19] were prospectively recruited for inclusion. This study aimed to evaluate patients with potential subtle microstructural alterations in the corpus callosum, and therefore all prior imaging studies were reviewed to include only patients whose corpus callosum demonstrated normal signal intensity without visible or active lesions on conventional MRI. Exclusion criteria for patients included: (1) contraindications for MRI, such as metallic implants or claustrophobia; (2) an inability to tolerate the MRI procedure without significant movement; (3) active MS relapse within the past 30 days. (4) history of alcohol misuse or comorbid neurological or major psychiatric disorders; (5) primary or secondary progressive MS; and (6) datasets degraded by artifact.

A total of 30 healthy controls were also recruited. Exclusion criteria for the control group included: (1) a diagnosis of multiple sclerosis or neuromyelitis optica spectrum disorder; (2) contraindications for MRI, such as metallic implants or claustrophobia; (3) any known brain or neurologic disorder, including, but not limited to: brain tumors, neurodegenerative conditions, epilepsy, or traumatic brain injury; (4) an inability to tolerate the MRI procedure without significant movement; and (5) datasets degraded by artifact. A consecutive sampling method was used to recruit RRMS patients from the Department of Medicine and Therapeutics, Prince of Wales Hospital, Shatin, (Hong Kong SAR), while convenience sampling was utilized to recruit age-matched healthy controls from the Hong Kong local community.

### 2.2. MRI Protocol

All MRI scans were acquired on a 3.0T Philips Ingenia Elition X scanner (Philips Medical Systems, Best, The Netherlands). The patient imaging protocol included conventional sequences comprising 3D T1-weighted fast field echo (FFE), 2D axial T2-weighted turbo spin echo (TSE), and 3D T2-FLAIR with fat suppression. In addition, a 3D T1-weighted FFE sequence with fat suppression was acquired following contrast administration (Dotarem, gadoteric acid 0.5 mmol/mL; Guerbet, Roissy CdG Cedex, France, 0.1 mL/kg). The parameters for the conventional sequences were as follows: 3D T1-weighted FFE (TR/TE = 7.6/3.5 ms; field of view = 250 × 250 mm; matrix = 228 × 208; slice thickness = 1.1 mm), 2D axial T2-weighted TSE (TR/TE = 2622/80 ms; field of view = 230 × 200 mm; matrix = 512 × 380; slice thickness = 2.5 mm; 50 slices), 3D T2-FLAIR TSE with fat suppression (TR/TE = 4800/340 ms; TI = 1650 ms; field of view = 250 × 250 mm; matrix = 224 × 223; slice thickness = 1.1 mm), and post-contrast 3D T1-weighted FFE with fat suppression (TR/TE = 26.4/2.3 ms; field of view = 230 × 230 mm; matrix = 256 × 256; slice thickness = 1.8 mm).

For quantitative T1rho imaging, a 3D T1rho-prepared TSE sequence was employed with TR/TE = 5000/25 ms, spin-lock frequency = 300 Hz, and spin-lock time (TSL) of 0, 10, 25, 45, and 75 ms. The acquisition included 80 sagittal slices with isotropic resolution (1.8 × 1.8 × 1.8 mm^3^) and incorporated B1 and B0 inhomogeneity compensation [20]. Controls underwent the same imaging protocol, except that the post-contrast T1-weighted FFE sequence was omitted.

### 2.3. Imaging Analysis

**Figure 1:**
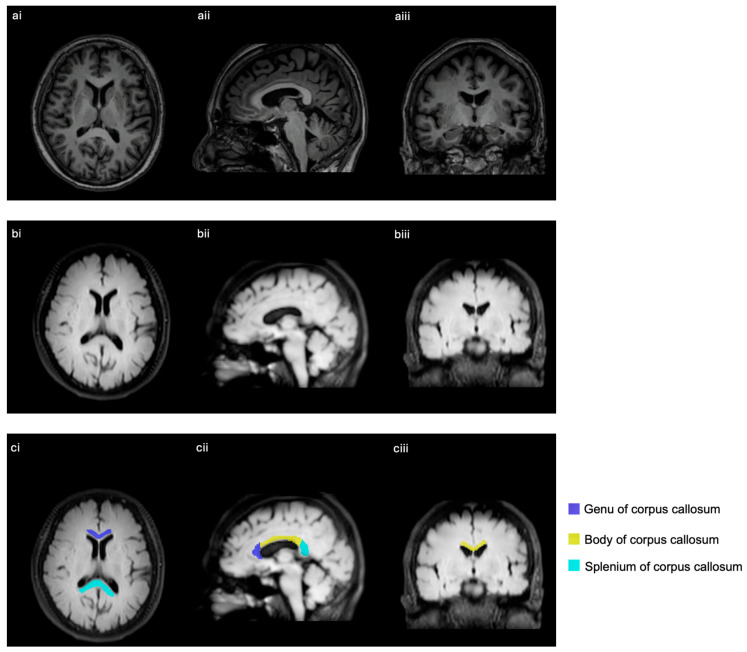
Illustrative examples of cross-sectional imaging and corpus callosum segmentation outcomes. Axial (**i**), sagittal (**ii**) and coronal (**iii**) slices presented of: (**a**) T1-weighted images; (**b**) corresponding T1rho images; (**c**) T1rho images with JHU-ICBM atlas-based corpus callosum segmentation overlay including the genu, body and splenium subregions.

The imaging analysis pipeline was implemented using the FMRIB Software Library (FSL, Oxford, UK; http://www.fmrib.ox.ac.uk/fsl, accessed on 10 September 2025) [21]. Following the co-registration of 3D T1 and T1rho images, the datasets underwent a series of standardized preprocessing steps. Brain extraction was performed using the Brain Extraction Tool (BET), to remove non-brain tissue, and spatial normalization was performed using a two-stage procedure. First, an affine (12 degrees-of-freedom) linear registration was applied with FLIRT. The resulting affine transform was then used in a non-linear registration with FNIRT, and the combined linear and non-linear transformations were subsequently applied to the T1rho maps. For segmentation of the corpus callosum, the Johns Hopkins University–International Consortium for Brain Mapping (JHU-ICBM) white matter atlas [22] was applied to the normalized probability maps to delineate the major white matter structures. As this atlas is defined in the standard space, the labels were transferred to the participant’s native space. The inverse non-linear transformation fields (inwarp) were calculated from the FNIRT deformation. These inverse fields were then applied to warp (nearest-neighbor interpolation) the JHU-ICBM atlas masks back into each participant’s native anatomical space. This atlas-based parcellation generated the segmentation masks of the corpus callosum subdivided into distinct anatomical regions (e.g., genu, body, and splenium), allowing for region-specific quantitative analyses of the corpus callosum (Figure 1). To ensure the accuracy of the corpus callosum segmentation, all masks were visually inspected and verified by a board-certified radiologist with over 10 years’ experience in neuroimaging; manual correction was performed where necessary, and any voxels extending beyond the anatomical boundaries of the corpus callosum were carefully removed.

### 2.4. Corpus Callosum Index

**Figure 2:**
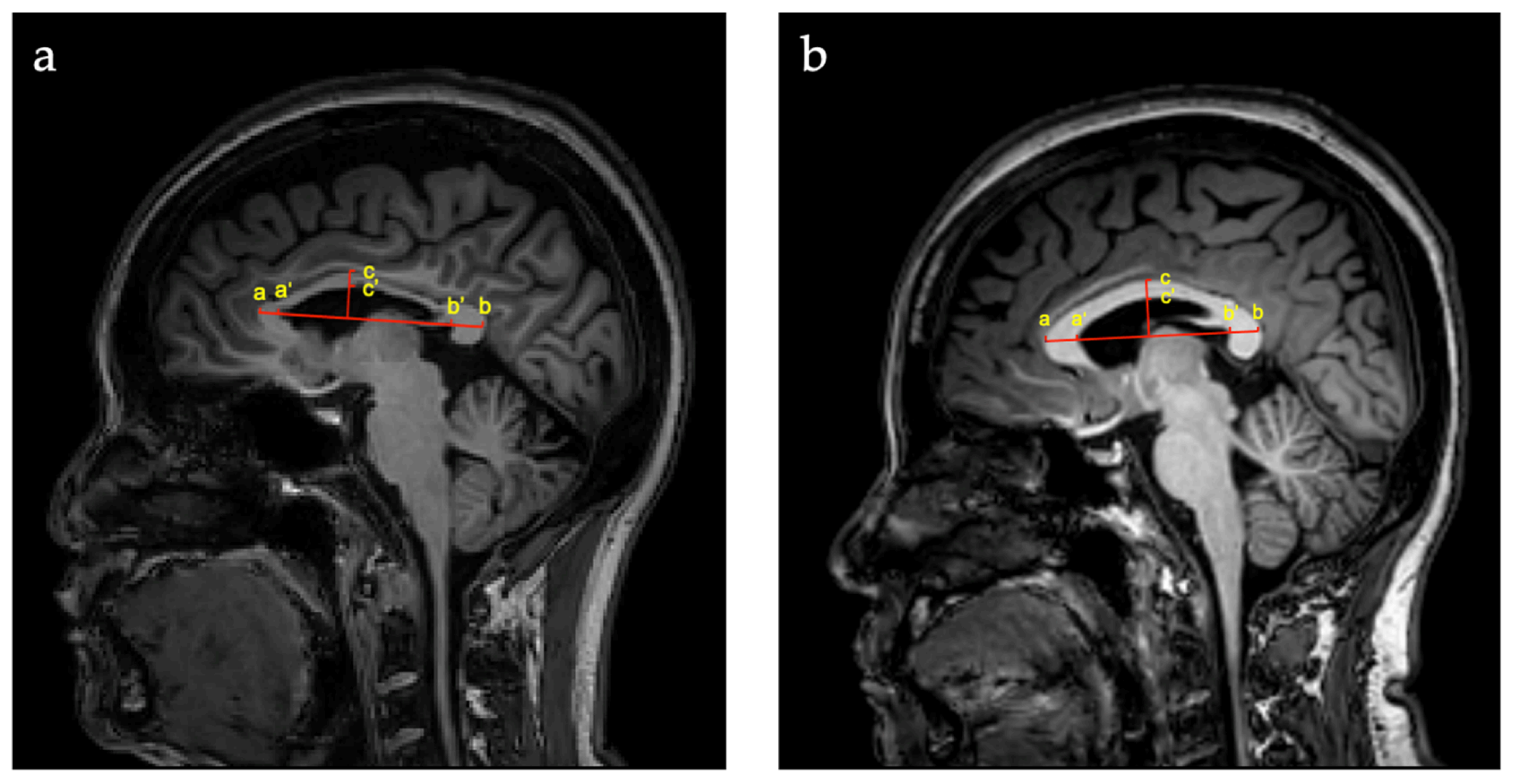
Measurement of the corpus callosum index (CCI) based on midsagittal T1-weighted images. Points a and a′, b and b′, and c and c′ were used to delineate the anterior, middle, and posterior segments of the corpus callosum, respectively. Each segment was then normalized to the maximum anteroposterior diameter of the corpus callosum (segment ab), yielding the CCI index. (**a**) Example of CCI measurement in a RRMS patient; (**b**) Example of CCI measurement in a control.

The CCI was measured using the method described by Figueira et al. from T1-weighted mid-sagittal slices of the corpus callosum [10]. A straight line was drawn along the greatest anteroposterior diameter of the corpus callosum, and the anterior (aa′), posterior (bb′), and middle (cc′) segments of the corpus callosum were then measured and normalized to the maximum anteroposterior diameter (ab) [10] (Figure 2). These values were summed to obtain the CCI as:

(1) $$CCI=\frac{aa^{\prime }+bb^{\prime }+cc^{\prime }}{ab}$$

The corpus callosum index (CCI) was measured twice, and the average of the two measurements was used for analysis.

### 2.5. T1rho Quantification

**Figure 3:**
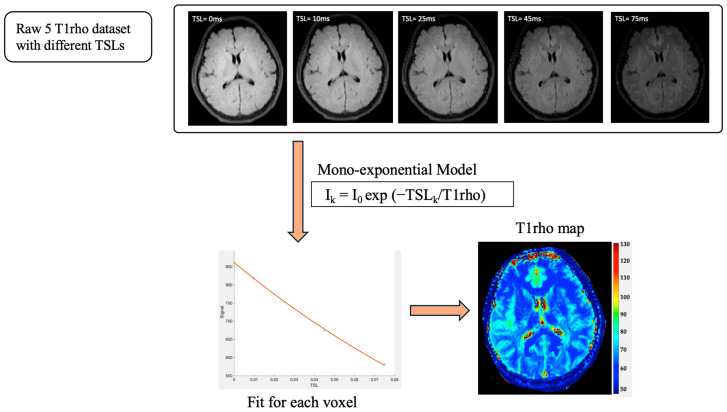
A simplified schematic illustration of the T1rho quantification process.

MATLAB R2021a (MathWorks, Natick, MA, USA) was employed for T1rho quantitative analysis. To reduce the adverse impact of noise and enhance the stability of pixel-wise fitting, a spatial filtering approach was applied prior to quantification [16]. At each pixel of the image, the intensity follows a mono-exponential decay model:

(2) I_k_ = I_0_ exp (−TSL_k_/T1rho)

where I_k_ is the image intensity for the kth T1rho-weighted image acquired with kth spin-lock duration (TSL_k_), and I_0_ is the image intensity when the spin-lock duration (TSL) is 0. T1rho-weighted images were smoothed by a Gaussian kernel with a 5 voxel × 5 voxel window, after which the data were fitted to Equation (2) using non-linear least squares fitting. The mean and standard deviation (SD) of pixel-wise T1rho values were then quantified (Figure 3).

### 2.6. Estimation of Total Brain White and Gray Matter Volume

Brain volume measurement was performed with the 3D T1-weighted image using SIENAX in FSL [23]. The process begins with skull stripping (BET) to remove non-brain tissue, followed by linear registration (FLIRT) to normalize the image to a standard template and account for individual head size differences. The brain-only image is then segmented with FAST into gray matter, white matter, and cerebrospinal fluid, with bias-field correction applied; lesion filling was not applied prior to SIENAX processing. Based on these segmentation results, SIENAX calculates total brain volume as well as tissue-specific volumes, providing reliable measures reports for each participant [23].

### 2.7. Estimation of Total Lesion Load

White matter lesions were segmented by the lesion growth algorithm (LGA) [24] as implemented in the lesion segmentation toolbox (LST) version 3.0.0 (www.statistical-modelling.de/lst.html, accessed on 10 September 2025) for Statistical Parametric Mapping (SPM12, London, UK; https://www.fil.ion.ucl.ac.uk/spm/software/spm12/ accessed on 10 September 2025). LGA was applied to segment T2-hyperintense lesions from a combination of T1 and FLAIR images [24]. The T1 image was first segmented into three tissue classes: CSF, gray matter, and white matter. These segmentation maps were then combined with FLAIR intensities to generate voxel-wise lesion belief maps. By thresholding these belief maps with a pre-defined threshold (kappa = 0.3), an initial binary lesion mask was obtained. This map was then subsequently expanded along voxels exhibiting hyperintensity on the FLAIR image. The final output was a lesion probability map, which was then binarized using a lesion probability threshold of 0.5 to yield the final lesion segmentation. All outputs were visually inspected and verified by a researcher with over 8 years of neuroimaging experience and subsequently verified by a radiologist with more than 10 years of research experience. The volume of all lesions was summed to estimate the total lesion load in mL for each participant.

### 2.8. Statistical Analysis

T1rho values, CCI, lesion load, and brain volumes were expressed as means ± standard deviations (SD). Data normality was assessed using the Shapiro-Wilk test. Comparisons of demographical and clinical data between the patient and control group were performed using the Mann–Whitney U test for non-parametric data and the independent samples *t*-test for parametric data. The chi-square test was applied to compare for differences in sex distribution between the MS patient group and the control group. Yates’ continuity correction was applied to the 2 × 2 contingency table to adjust for the discrepancy between the discrete nature of the observed data and the continuous approximation assumed by the chi-square test.

To evaluate regional differences in T1rho values within the corpus callosum across the genu, body, splenium, and the whole corpus callosum between patients and controls, as well as between different subregions for patients and controls, paired *t*-tests and Wilcoxon signed-rank tests were used. To account for the increased risk of a Type I error associated with multiple statistical testing, Bonferroni correction was applied to pairwise comparisons. Statistical significance was defined as a *p*-value less than 0.05.

Pearson’s and Spearman’s correlation coefficients were used to evaluate the correlation between T1rho values and EDSS scores, disease duration, lesion load, and CCI. Confidence intervals (CIs) of 95% for correlation and regression coefficients were estimated using bootstrap resampling with 1000 iterations. All statistical analyses were performed using MedCalc (version 20.100; MedCalc Software, Ostend, Belgium).

## 3. Results

Participant demographics are presented in Table 1. A total of 44 patients were initially recruited, of which 14 were excluded (8 due to motion-related artifacts; and a further 6 due to the presence of confluent corpus callosum lesions). The final study population included 30 RRMS patients (37.93 ± 8.23 years; 24 females, 6 males), and 30 controls (37.73 ± 9.92 years; 18 females, 12 males). There were no significant differences in age or sex distribution between the patient and the control group.

Total white matter volume (683.42 ± 39.97 mL) and whole brain volume (1437.59 ± 60.17 mL) were significantly lower in MS patients than in controls (*p* < 0.01), while gray matter volume in MS patients (754.18 ± 32.33 mL) was comparable to that of the control group (742.04 ± 35.43 mL; *p* = 0.06).

**Figure 4:**
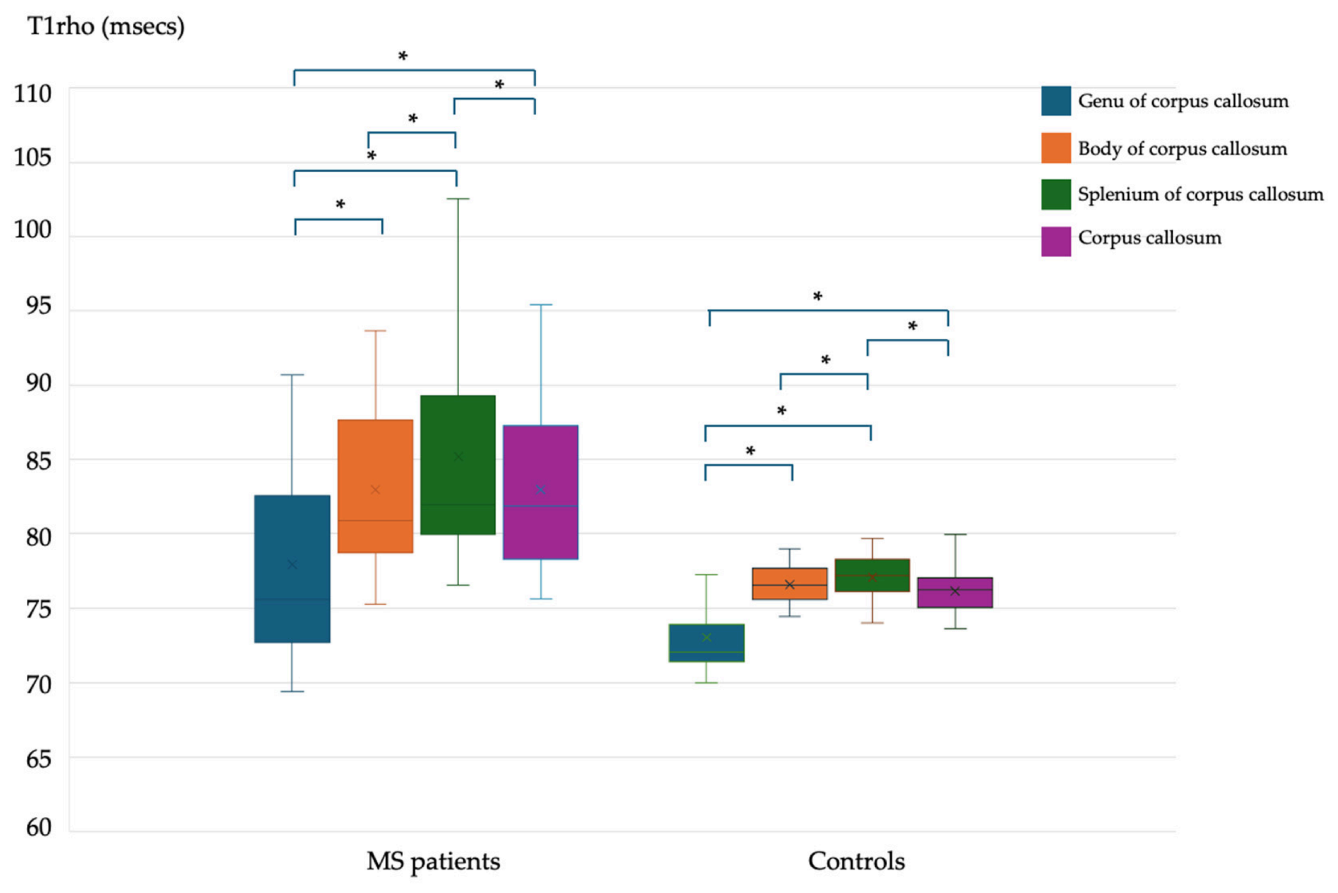
Comparison of T1rho values across corpus callosum subregions in both MS patients and controls. The genu consistently exhibited significantly lower T1rho values relative to the body, splenium, and the whole corpus callosum in both groups. Bonferroni correction was applied to pairwise comparisons. * Significant results (*p*-value < 0.05).

T1rho values within the corpus callosum were consistently elevated in patients compared to controls. Significant increases were observed across all major subregions, including the genu, body, and splenium, as well as in measurements of the whole corpus callosum (Table 2). Among MS patients, T1rho values in the genu of the corpus callosum (77.95 ± 6.72 ms) were the lowest of all subregions and significantly reduced compared with the body (82.97 ± 5.27 ms), splenium (85.17 ± 7.96 ms), and the whole corpus callosum (82.97 ± 5.53 ms). A similar regional pattern was observed in controls, with the genu (73.03 ± 3.11 ms) showing lower T1rho values relative to the body, splenium, and whole corpus callosum (Figure 4).

T1rho values in the body (correlation coefficient = 0.392, *p* = 0.032), splenium (correlation coefficient = 0.375, *p* = 0.041), and whole corpus callosum (correlation coefficient = 0.408, *p* = 0.025) demonstrated a positive association with disease duration time (Table 3). Moreover, T1rho values across all corpus callosum regions (genu, body, splenium, and whole corpus callosum) were positively correlated with lesion load (r, 0.726–0.810, *p* < 0.001). T1rho values in these regions exhibited a negative correlation with the CCI (r, −0.731–−0.640, *p* < 0.001), indicating that higher T1rho values were associated with greater callosal atrophy. T1rho values in the body (r = −0.464, *p* = 0.010) and whole corpus callosum (r = −0.399, *p* = 0.029) showed a negative correlation with total white matter volume in MS patients. 

The CCI was negatively correlated with lesion load (r = −0.739, *p* < 0.001), disease duration time (r = −0.421, *p* = 0.021), and positively correlated total white matter volume (r = 0.487, *p* = 0.006) (Table 4). Total white matter volume was negatively associated with lesion load (r = −0.541, *p* = 0.002). Whole brain volume was positively correlated with total white matter (r = 0.778, *p* < 0.001) and total gray matter (r = 0.636, *p* < 0.001) volumes. EDSS was positively correlated with disease duration time (r = 0.365, *p* = 0.048), but no significant correlations between T1rho values and EDSS scores were found.

## 4. Discussion

In this study, T1rho values within the corpus callosum, including the genu, body, splenium, and whole corpus callosum were consistently higher in patients with MS compared to controls. These results are consistent with previous studies in MS, which have demonstrated T1rho values to be significantly elevated in the white matter of MS patients compared to healthy controls, including within regions of NAWM [17]. Previous studies in MS have demonstrated diffuse axonal injury and microglial activation within the NAWM, disrupting the microstructural and macromolecular environment of brain tissue [25]. These pathological changes likely contribute to elevated T1rho values and produce detectable quantitative changes even in areas that appear normal on MRI [17,26]. The findings from this study confirm the involvement of the corpus callosum in MS and demonstrate the potential of T1rho imaging as a promising tool for detecting such microstructural changes.

The regional pattern of lower T1rho values in both patients and controls represents an interesting phenomenon that warrants further consideration. Hofer et al. previously reported regional differences in T1 relaxation times across the corpus callosum [27]. In the study, T1 value was likewise lowest in the genu of the corpus callosum [27]. Diffusion MRI studies by Horowitz et al. demonstrated a systematic variation in axonal diameters across the corpus callosum, with narrower axons in the genu, larger axons in the body, and intermediate diameters in the splenium [28]. This anatomical variability in fiber architecture was further supported by electron microscopy findings in a study reported by Aboitiz et al. [29], in which large-diameter fibers reach peak density in the mid-posterior corpus callosum and show an increase in density in the posterior pole. Taken together, these findings suggest that the regional variation in T1rho values observed in our study may reflect underlying differences in axonal diameter and density across callosal subregions, potentially explaining the lower T1rho values observed in the genu. Further studies are needed to clarify the precise structural and biochemical mechanisms underlying these regional T1rho variations.

In this study, T1rho values were positively associated with disease duration and lesion load. The relapsing nature of MS likely contributes to recurrent inflammatory activity that leads to cumulative alterations related to ongoing demyelination and neurodegeneration [30]. T1rho changes may reflect macromolecular changes in this spectrum of underlying pathological processes, including inflammatory changes, irreversible axonal injury, demyelination, or a combination of these mechanisms. Such changes may drive the progressive increase in T1rho values compared with longer disease course. Thus, a greater lesion burden may correspond to increased tissue damage, resulting in higher overall T1rho relaxation time. However, in the absence of direct histopathological correlation, currently, T1rho should be interpreted as a composite marker of tissue microstructural abnormality rather than a pathology-specific biomarker. This limitation is consistent with prior quantitative MRI studies, where overlapping biological substrates contribute to signal changes [14,17].

Our results revealed CCI to be significantly negatively correlated with disease duration and lesion load. The accumulation of lesions and diffuse white matter damage may also accelerate structural loss within the corpus callosum. CCI has been previously suggested to be not only a metric of corpus callosal volume but also an indicator of overall general brain volume [11,31,32]. However, in this study, CCI was well correlated with total white matter volume, but not whole brain volume. Figueira et al. also previously reported that CCI correlates with EDSS in MS [10]. However, no significant association between T1rho values or CCI and EDSS was observed in this study. Because EDSS is weighted primarily toward motor and ambulatory function [33,34], it may underestimate other aspects of disease burden, including cognitive impairment [35] and subclinical tissue loss detectable on MRI. The absence of this imaging-clinical correlation in this study therefore does not diminish the biological relevance of T1rho abnormalities, but rather reflects the recognized limitation of EDSS as a global outcome measure to the full spectrum of MS-related tissue injury. Although disease modifying therapies (DMTs) reduce inflammatory activity and slow the progression of clinical disability [36,37,38], they may not entirely prevent neurodegeneration or cerebral atrophy [39]. In our results, patients with MS exhibited significantly greater atrophy of white matter and brain volume compared with healthy controls, which is consistent with findings reported in the previous literature.

The findings from this study may suggest that T1rho could offer information that is complementary to conventional MRI and established structural measures. Unlike T2-weighted and FLAIR imaging, which depict macroscopic lesions, T1rho appears sensitive to diffuse microstructural change within the corpus callosum. While CCI quantifies structural atrophy, T1rho may reflect the microstructural integrity of the tissue, and their correlation suggests that both may reflect cumulative disease burden.

This study has several limitations that should be acknowledged. First, T1rho values may vary across different MRI scanners, including MRI scanners of varying field strengths and acquisition protocols, which could introduce variability in quantitative measurements. However, in this study, all patients and controls were scanned using the same scanner and with the same acquisition protocol, minimizing such variability. We acknowledge that the exclusion of patients with focal callosal lesions may have introduced potential selection bias. Consequently, our findings likely reflect callosal microstructural changes in patients with mild-to-moderate RRMS, and may not be generalizable to those with severe disease. Meanwhile, while noting that stable DMT use was not a primary driver of our results, its subtle effects on tissue microenvironment cannot be entirely ruled out. Lastly, given the limited sample size, our analysis was limited to bivariate correlations without multivariable adjustment. Given the potential for additional potential confounding factors, the observed associations should therefore be regarded as exploratory. We note, however, that the patient and control groups were matched for age and sex, minimizing the influence of these demographic factors on the comparison.

## 5. Conclusions

In summary, T1rho values in the genu, body, and splenium of the corpus callosum of patients with MS were significantly elevated compared with healthy controls. T1rho values in the corpus callosum demonstrated significant correlations with the lesion load, disease duration, and the CCI, suggesting cumulative microstructural alterations accrued over the disease course, linked with disease burden and CC atrophy. Corpus callosum T1rho values may have potential value as a complementary imaging biomarker for disease progression in MS.

## Figures and Tables

**Table 1:** Demographics and clinical characteristics of study participants.

	Patients (*n* = 30)	Controls (*n* = 30)	*p*-Value
Age, years (Mean ± SD)	37.93 ± 8.23	37.73 ± 9.92	0.68
Gender	24 females, 6 males	18 females, 12 males	0.23
EDSS score (Mean ± SD)	1.68 ± 1.89	N/A	N/A
Disease duration, years (Mean ± SD)	9.79 ± 7.74	N/A	N/A
CCI (Mean ± SD)	0.35 ± 0.08	0.42 ± 0.04	<0.01 *
Lesion load, mL (Mean ± SD)	8.89 ± 9.60	N/A	N/A
Total gray matter volume, mL (Mean ± SD)	754.18 ± 32.33	742.04 ± 35.43	0.06
Total white matter volume, mL (Mean ± SD)	683.42 ± 39.97	733.43 ± 27.87	<0.01 *
Whole brain volume, mL (Mean ± SD)	1437.59 ± 60.17	1475.47 ± 42.12	<0.01 *

Bonferroni correction was applied to pairwise comparisons. * Significant results (*p*-value < 0.05).

**Table 2:** T1rho values in the corpus callosum.

	Patients (*n* = 30)	Controls (*n* = 30)	*p*-Value	Cohen’s *d*
T1rho value (ms)				
Genu of corpus callosum	77.95 ± 6.72	73.03 ± 3.11	<0.01 *	0.94
Body of corpus callosum	82.97 ± 5.27	76.57 ± 1.24	<0.01 *	1.68
Splenium of corpus callosum	85.17 ± 7.96	77.07 ± 1.50	<0.01 *	1.41
Whole corpus callosum	82.97 ± 5.53	76.16 ± 1.48	<0.01 *	1.68

Bonferroni correction was applied to pairwise comparisons. * Significant results (*p*-value < 0.05).

**Table 3:** Correlation between corpus callosum T1rho values and clinical and imaging markers in patients with MS.

	Age (Years)		EDSS Score		Disease Duration Time (Years)		Lesion Load (mL)		Corpus Callosum Index (CCI)		Total Gray Matter Volume (mL)		Total White Matter Volume (mL)		Whole Brain Volume (mL)	
	Correlation Coefficient 95% CI)	*p*-Value	Correlation Coefficient (95% CI)	*p*-Value	Correlation Coefficient (95% CI)	*p*-Value	Correlation Coefficient (95% CI)	*p*-Value	Correlation Coefficient (95% CI)	*p*-Value	Correlation Coefficient (95% CI)	*p*-Value	Correlation Coefficient (95% CI)	*p*-Value	Correlation Coefficient (95% CI)	*p*-Value
T1rho Value (ms)																
Genu of corpus callosum	0.177 (−0.196–0.505)	0.350	0.249 (−0.122–0.559)	0.184	0.276 (−0.094–0.578)	0.140	0.728 (0.499–0.862)	<0.001 *	−0.727 (−0.861– −0.497)	<0.001 *	0.313 (0.054–0.605)	0.093	−0.350 (−0.631–0.012)	0.058	−0.040 (−0.394–0.325)	0.835
Body of corpus callosum	0.140 (−0.232–0.476)	0.461	0.009 (−0.353–0.368)	0.964	0.392 (0.037–0.659)	0.032 *	0.781 (0.585–0.890)	<0.001 *	−0.640 (−0.813– −0.363)	<0.001 *	0.341 (−0.022–0.625)	0.065	−0.464 (−0.706– −0.124)	0.010 *	−0.134 (−0.471–0.238)	0.481
Splenium of corpus callosum	0.122 (−0.250–0.462)	0.522	−0.041 (−0.395–0.324)	0.830	0.375 (0.017–0.648)	0.041 *	0.726 (0.496–0.861)	<0.001 *	−0.644 (−0.815– −0.370)	<0.001 *	0.358 (−0.002–0.636)	0.052	−0.246 (−0.557– 0.125)	0.190	0.051 (−0.315–0.404)	0.789
Whole corpus callosum	0.169 (−0.204–0.499)	0.372	0.068 (−0.300–0.418)	0.722	0.408 (0.056–0.670)	0.025 *	0.810 (0.634–0.906)	<0.001 *	−0.731 (−0.864– −0.504)	<0.001 *	0.366 (0.006–0.641)	0.047 *	−0.399 (−0.664–−0.046)	0.029 *	−0.069 (−0.419–0.299)	0.718

* Significant results (*p*-value < 0.05).

**Table 4:** Correlation between clinical and imaging markers in patients with MS.

	EDSS Score		Disease Duration Time (Years)		Lesion Load (mL)		Corpus Callosum Index (CCI)		Total White Matter Volume (mL)		Total Gray Matter Volume (mL)		Whole Brain Volume (mL)	
	Correlation Coefficient (95% CI)	*p*-Value	Correlation Coefficient (95% CI)	*p*-Value	Correlation Coefficient (95% CI)	*p*-Value	Correlation Coefficient (95% CI)	*p*-Value	Correlation Coefficient (95% CI)	*p*-Value	Correlation Coefficient (95% CI)	*p*-Value	Correlation Coefficient (95% CI)	*p*-Value
EDSS score	–	–	–	–	–	–	–	–	–	–	–	–	–	–
Disease duration time (years)	0.365 (0.005–0.641)	0.048 *	–	–	–	–	–	–	–	–	–	–	–	–
Lesion load (mL)	0.183 (0.189–0.510)	0.332	0.308 (−0.059–0.601)	0.098	–	–	–	–	–	–	–	–	–	–
Corpus callosum index (CCI)	−0.186 (−0.512–0.187)	0.326	−0.421 (−0.616–−0.036)	0.021 *	−0.739 (−0.868- −0.517)	<0.001 *	–	–	–	–	–	–	–	–
Total white matter volume (mL)	−0.348 (−0.630–0.014)	0.059	−0.336 (−0.486–−0.220)	0.070	−0.541 (−0.754– −0.224)	0.002 *	0.487 (0.153–0.721)	0.006 *	–	–	–	–	–	–
Total gray matter volume (mL)	−0.211 (−0.531–0.161)	0.262	−0.152 (−0.578–0.095)	0.421	0.192 (−0.180–0.517)	0.308	−0.203 (−0.525–0.170)	0.282	0.086 (−0.286–0.433)	0.651	–	–	–	–
Whole brain volume (mL)	−0.304 (−0.598–0.064)	0.103	−0.239 (−0.552–0.133)	0.204	−0.271 (−0.575–0.099)	0.148	0.205 (−0.167–0.527)	0.276	0.778 (0.580–0.889)	<0.001 *	0.636 (0.357–0.810)	<0.001 *	–	–

* Significant results (*p*-value < 0.05).

## Data Availability

The data supporting this study are not publicly available due to institutional regulations and patient privacy considerations.

## Abbreviations

The following abbreviations are used in this manuscript:

BET	Brain extraction tool
CCI	Corpus callosum index
CCV	Corpus callosum volume
CI	Confidence interval
DMTs	Disease modifying therapies
EDSS	Expanded disability status scale
FFE	Fast field echo
FSL	FMRIB software library
LGA	Lesion growth algorithm
MS	Multiple sclerosis
NAWM	Normal-appearing white matter
JHU-ICBM	Johns Hopkins University–International Consortium for Brain Mapping
RF	Radiofrequency
ROI	Region of interest
RRMS	Relapsing-remitting multiple sclerosis
SL	Spin-lock
SPM	Statistical parametric mapping
TSE	Turbo spin echo
TSL	Spin-lock time
